# Supplementary material for: Emergent dynamics due to chemo-hydrodynamic self-interactions in active polymers
Source: Nat Commun. 2024 Jun 8;15:4903. doi: 10.1038/s41467-024-49155-7 (PMC11162426; doi:10.1038/s41467-024-49155-7)
Supplement: Supplementary file 1 — Supplementary Information [file 41467_2024_49155_MOESM1_ESM.pdf]

Supplementary Information
Emergent dynamics due to chemo-hydrodynamic self-interactions in active polymers

Manoj Kumar,[1,*] Aniruddh Murali,[1] Arvin Gopal Subramaniam,[2] Rajesh Singh,[2,†] and Shashi Thutupalli[1,3,‡]

[1] *Simons Centre for the Study of Living Machines, National Centre for Biological Sciences, Tata Institute of Fundamental Research, Bangalore, India*

[2] *Department of Physics, Indian Institute of Technology, Chennai, India*

[3] *International Centre for Theoretical Sciences, Tata Institute of Fundamental Research, Bangalore, India*

[*] manojk@ncbs.res.in

[†] rajesh.singh@physics.iitm.ac.in

[‡] shashi@ncbs.res.in

I. EXPERIMENTAL SECTION

A. Droplet Fabrication

The 5CB LC monodisperse emulsion droplets were produced using a 3-channel microfluidic device, as shown in the schematic (Fig. S1**A**). The microfluidic flow-focus device was fabricated using a previously reported protocol [**?**]. In a 10:1 ratio (w / w), we thoroughly mixed PDMS (polydimethylsiloxane) with a SYLGARD^{TM} 184 silicon elastomer curing agent. To remove the air bubbles formed during mixing, the mixture was degassed in a desiccator for 1 h. The mixture was then gently poured into a silicon wafer mold to avoid bubble formation and was cured for 5 h at 70 °C. We then used a PDC-002 plasma cleaner to clean the PDMS mold and a glass slide for 5 min (HARRICK PLASMA). After plasma treatment, the PDMS mold was attached to the glass slide and heated to 70 °C for 1 min. Because the aqueous phase is a continuous phase in our setup, the microfluidic device channels were made hydrophilic by flowing a 1% aqueous solution of polyvinyl alcohol (PVA) through the channels. With a 0.75 mm diameter puncture, we make two holes for the inlet and one for the outlet. Polyethylene tubing (BTPE-50) was used to connect the device holes to reservoir liquids (oil and aqueous phase) (purchased from Instech Laboratories, Inc.). The inner and outer diameters of the tubing are 0.58 and 0.97 mm, respectively, and the tubes were cut to equal lengths (50 cm each). The 5CB LC oil phase and the aqueous phase were separated into separate 1 mL plastic syringes and injected into the microfluidic device channels using a syringe pump (NEMESYS low pressure syringe pump). The flow rates for the aqueous phase were kept at 10 $\mu L/min$ and the flow rates for the oil phase (5CB LC) were kept at 0.1 $\mu L/min$. We were able to produce millions of emulsion (5CB LC) droplets with a diameter 50 μm, which we collected in a 0.25 (w/v)% SDS solution. These droplets remained stable for months.

B. Flow Cell Fabrication

We designed a quasi two-dimensional flow cell to perform all microscopic measurements on active droplets and their assemblies. We used double-sided adhesive tape (3M, USA; product no. 82601) with a thickness of 10 μm to make a flow cell. We used 5x10 μm of the adhesive tape in a layer-by-layer fashion to create a flow cell with a height of h$\sim$ 50 μm using a glass slide and a cover slip (schematic shown in Fig. S1**E**).The two ends of the flow cell were kept open for droplet injection later; both ends were sealed with nail polish to prevent external flows while measuring the flow fields, chemical fields and dynamics of the active assemblies.

C. Image analysis

We record the images using a microscope, each image is 2048 x 2048 pixels in size (Fig. S2**A**), and the images were cropped as shown in Fig. S2**B**. To improve the contrast of the droplets against the background, a background image was generated by averaging all the images in the sequence and subtracting it from each frame (Fig. S2**C**). This step also helped to remove non-moving objects from the field of view. The resulting images were processed using custom code in Matlab. First, a region of interest was defined around the interested polymer chains. The droplets were then detected using the Imfindcircles function in Matlab (Fig. S2**D**). A least square tracking algorithm, tailored to the current experiment data, was used to stitch the detected centers in successive frames and form trajectories. To convert the trajectories from pixels to micrometers, the correct conversion factor was applied, and the scales were adjusted accordingly. At t=0, the trajectories were transformed to ensure that the center of mass of the polymer chain began at [0, 0]. Using these trajectories, it was possible to calculate the speed, radius of curvature, and bond angles of the polymer chains.

II. DATA ANALYSIS

A. Speed calculation

Videos were recorded for a period of 10 minutes at a frame rate of 1 frame/s to extract trajectory data for the self-propelling active polymer chains. The V_{CM} velocities of the chains were calculated by measuring the displacement of their center of mass over a 10-frame interval (10 s). Previous studies have shown that the speed of active droplets decreases as their size decreases over time. However, the velocities recorded for the 10-minute period did not show any sign of a decrease in velocity for the cases of dimer, pentamer, and hexamer, as illustrated in Fig. S3**A**.

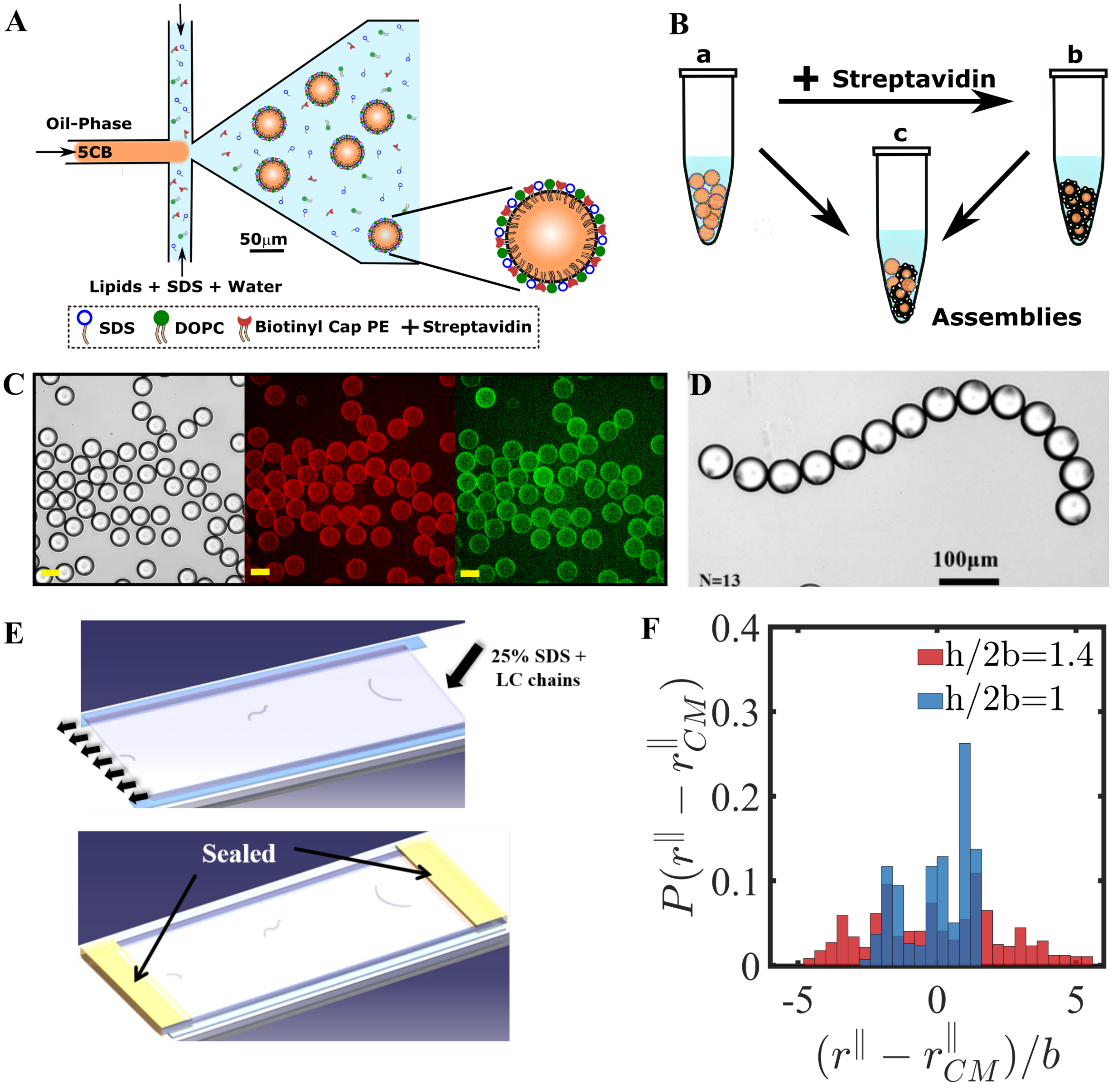

Figure S1. **(A)** Microfluidic set up for the production of monodisperse 5CB LC oil emulsion droplets stabilised by the aqueous solution of lipids (DOPC, Biot-Cap-PE)-surfactant (SDS). **(B)** Schematic shows the protocol for mixing the 5CB emulsion droplets to make assemblies by modifying the droplets surface with steptavidin (a to b), thereby stitching with biotinylated 5CB emulsion droplets (by mixing "a" and "b" in "c"), and resulting in assemblies. **C** Bright field microscipy image of 5CB emulsion droplets (*left panel*); fluorescence microscopy image confirms the presence of lipids in the surfactant-lipids monolayer (*middle panel*, Liss-Rhod-PE, λ_e 570 nm); fluorescence image confirms biot-Cap-PE lipids in the monolayer (*right panel*, streptavidin with Alexa-488, λ_e 488 nm), scale bar: 50 μm . **(D)** The chain of maximum length $N = 13$ is obtained in a very low fraction without any branching.**(E)** Design for a quasi two-dimensional flow cell of height h $\sim 50\mu m$ fabricated using double-sided adhesive tape of thickness 10 μm (stacking together 5-layers on the glass slide), glass slide, and a glass cover slip with open inlets and outlets to inject and remove the sample, **(top)** the sealed ends of the flow cell. **(F)** The distance from the center of mass in directions parallel $r^{\parallel} - r^{\parallel}_{CM}$ to the motion of centre of mass is plotted.

As the chain size increases ($N = 1$ to $N = 13$), the probability of finding the chain decreases exponentially, resulting in a varying number of realizations (ranging from 100 to 5) for each chain size. To obtain reliable speed measurements, the velocities plotted in Fig. 4**B** were averaged over many different realizations, with the mean speed and the mean standard deviation of the speed for each individual run, and we have shown a case of tetramer in Fig. S3**B**. The range of average velocities and their standard deviation appeared to be contained within a small region, indicating that the various realizations of self-propelling active polymer chains exhibit similar velocities.

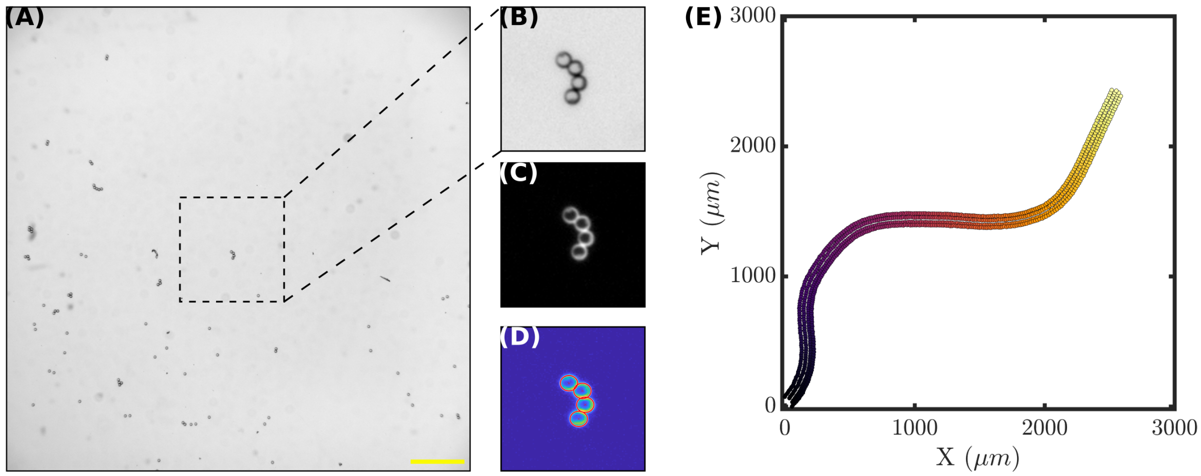

Figure S2. Image Analysis: **(A)** Microscopic image obtained after slicing up the video, **(B)** Zoomed in view of tetramer; **(C)** Background subtracted image; (D) Droplet center detection using imfindcircles function; **(E)** Trajectories of the detected droplets.

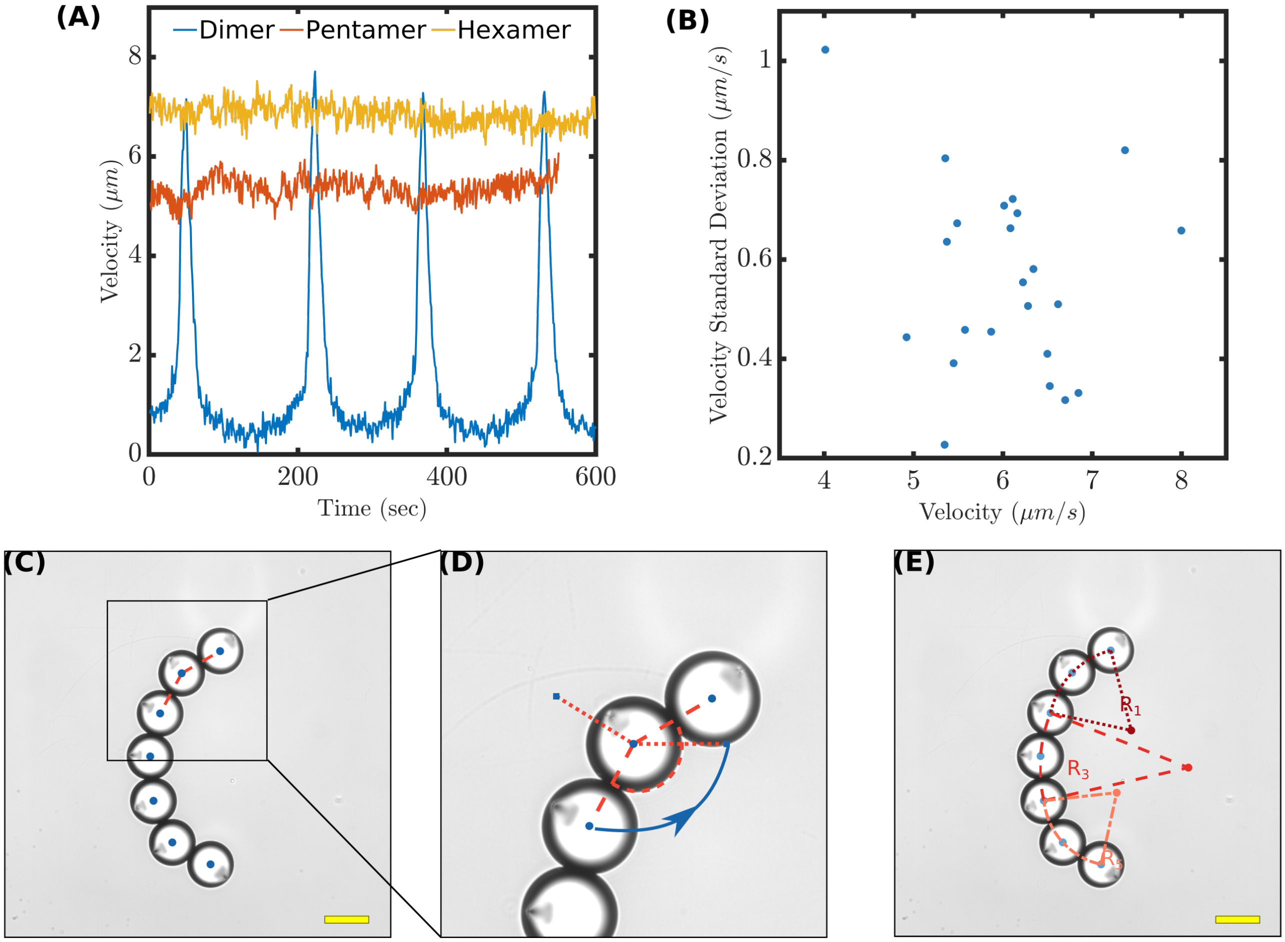

Figure S3. Velocity: **(A)** V_{cm} vs t for dimer(blue), pentamer(orange), hexamer(Red); **(B)** Scatter plot of $V_{cm}(\mu m/s)$ vs $\sigma(V_{cm})$ for tetramer (number of realisation 19); Bond Angle: **(C)** 32X magnification Image of septamer with Centers Detected (Scale Bar = 50 μm); **(D)** Zoomed in view of a triplets of droplet, showing bond angle (dashes original angle, dots rotated frame of reference); Radius of curvature: **(E)** Radius of curvature for three sets of triplets (1-2-3, 3-4-5, 5-6-7);

B. Bond Angle

To calculate the bond angles of the self-propelling active polymer chains, we used their trajectory data and processed it using a custom code optimized for the experimental data. Before passing the trajectory data through the code, the centers of the droplets in the chain were sorted such that the leftmost droplet was the first one in the chain.

Next, we rotated the coordinate axis of the triplet section (as shown in the box in Fig. S3**C**) around the middle droplet, so that the centers of the middle and final droplets aligned with the X-axis, as illustrated in Fig. S3**D** (dotted line). The angle subtended by the first droplet with respect to the X-axis corresponds to the bond angle of the triplet. We used the *atan2d* function to calculate all bond angles, with angles ranging from $-\pi$ to π, as shown in Fig. S5**A**. The calculation error for the bond angles was found to be 0.6 degrees.

C. Radius of Curvature

To calculate the radius of curvature, trajectories processed before using image analysis were used. As discussed in the previous section II B, the sorted droplet centers were used to calculate the curvature. Three consecutive droplets (triplets) centers were passed through the function for a given frame that calculates the center and radius of the circle passing through the 3 points (Fig. S3**E**). These radii were calculated for each possible triplet of consecutive droplets in the chain and averaged to obtain the mean radius for particle frame (Fig. S3**E** shows 3 of the 5 possible curvature radii). The calculated mean radius of curvature is then averaged over time to get the time-averaged radius of curvature. The radius of curvature calculated for the droplets in middle of the chain (i.e., all triplets that do not include the edge droplet) has an extremely high radius of curvature.

D. Chemical Field

Chemical field images were obtained from videos recorded using fluorescence microscopy, and the chemical field was visualized using an oil-soluble fluorescent dye (Fig. S4**A**). The videos recorded using fluorescent microscopy were pre-processed by capping the maximum and minimum fluorescence intensities. The images were processed through a custom image processing code (Section I C) as discussed above, to detect droplet centers. Using the detected centers, all the frames in the video were transformed and rotated so that they lie on top of each other. A mask was applied over the droplets for each frame to reduce the intensity in the masked region to 0. Individual frames were normalized with the maximum intensity being 1 and the minimum being 0. The frames were averaged and passed through a Gaussian filter to increase the signal-to-noise ratio for an improved chemical field image (Fig. S4**B**).

E. Particle Image Velocimetry

Streamlines were obtained using PIVlab [2]. Videos of active chains with tracer particles (polystyrene beads, $\sim 0.5\ \mu m$) were recorded using fluorescence microscopy (10 frames/s) (Fig. S4**C**). The droplets centers were detected using the custom code mentioned in section I C. A mask was created according to the droplet size and center based on the detection in each frame. The mesh size and time points were selected so that we have enough displacement for a good correlation. A mesh size of 160 and two steps of 80 and 40 were used, and every 5th frame was selected for the PIV analysis. Masks were applied to each frame, and then the analysis was carried out in PIVlab (Fig. S4**D**). The data, i.e., the x-component velocity (u), the y-component velocity (v), and the x and y (of the mesh), are exported and used in custom code to perform a time-average PIV. Similarly to the chemical field, the images were transformed so that they lie on top of each other after time-averaging the corresponding velocity.

F. Flow Trace

FlowTrace plugin in ImageJ [3] was used to trace the flows from the same set of images used in the PIV analysis. The centers of the droplets were detected using the same code as used in the PIV analysis (Section II E). A mask was created to reduce the brightness of the droplet to "0" and was applied to all sets of images. Once the mask was applied, the set of images was passed through the FlowTrace plugin. The flow-trace trails were calculated using a step of 30 frames. Droplets were added back to the final flow-trace image by inverting the mask. The flow trace image of a linear assembly can be seen in Fig. S4**E**.

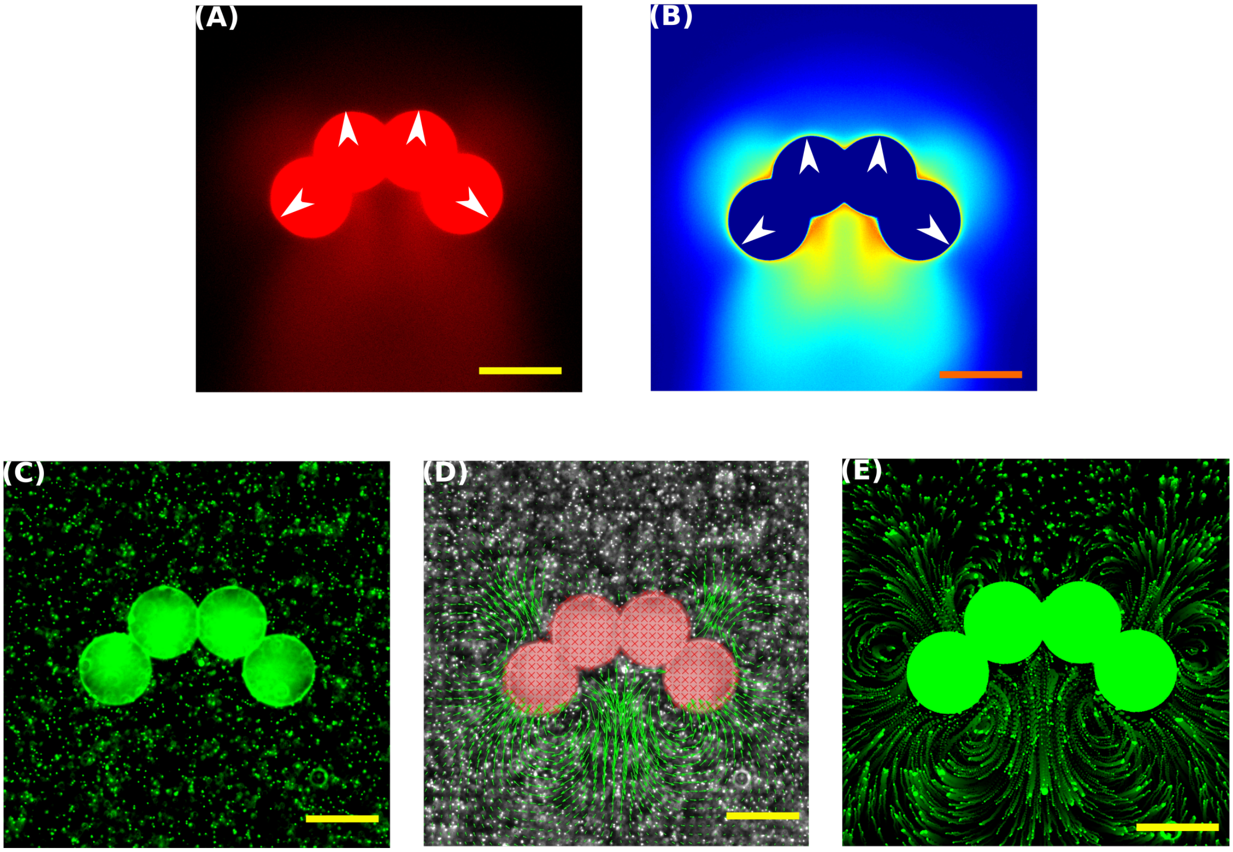

Figure S4. Chemical fields and hydrodynamic flow fields of a tetramer measured using a fluorescence microscopy. The chemical field is visualised using an oil soluble (Nile red) dye (excitation/emission maxima ∼552/636 nm) and the flow fields were visualised using a 0.5 μm tracer particles (FluoSpheres Carboxylate-modified microspheres), yellow-green fluorescence (excitation/emission maxima ∼505/515 nm) (scale: 50 μm). **(A)** The chemical field of a tetramer; **(B)** shows the time-averaged chemical field; **(C)** Fluorescence image of tetramer with fluorescent tracer particles (scale: 50 μm); **(D)** PIV analysis shows the flows around the tetramer; **(E)** The flows around the tetramer are visualised using a FloTrace plugin in ImageJ

.

III. THEORY AND SIMULATIONS

A. Computing chemical interactions in active chains

We model the ith active droplet as a colloid particle centered at $\boldsymbol{R}_i$, confined to move in two-dimensions, which self-propels with a speed v_s, along the directions $\boldsymbol{e}_i = (\cos\theta_i, \sin\theta_i)$. The direction of the particle, given by the angle θ, change due to coupling to a phoretic field c. The position and orientation of the ith particle is updated as per Eq(1) of the main text. The particles interact through the chemical field c that they produce: which follows the equation

$$\frac{\partial c(\boldsymbol{r},t)}{\partial t} = D_c \nabla^2 c(\boldsymbol{r},t) + \sum_{i=1}^{N} \lambda_e \delta(\boldsymbol{r} - \boldsymbol{R}_i). \tag{S1}$$

Here D_c is the diffusion coefficient of the filled micelles and λ_e is emission constant of the micelles. It should be noted that we model the droplet as a point particle. Using the above, we can solve for $\boldsymbol{\mathcal{J}}_i$ as:

$$\boldsymbol{\mathcal{J}}_i = \frac{2\lambda_e}{\pi^{d/2} H} \sum_{\substack{j=1 \\ j\neq i}}^{N} \int_{-\infty}^{t} dt' \, \frac{\boldsymbol{R}_i - \boldsymbol{R}_j(t')}{(4D_c|t-t'|)^{1+\frac{d}{2}}} \, e^{\frac{-[\boldsymbol{R}_i - \boldsymbol{R}_j(t')]^2}{4D_c|t-t'|}}. \tag{S2}$$

Here H is the height of the Hele-Shaw cell. In the above, we have ignored the fluid flow $\mathbf{v}$. As we show below, we can capture the essential features of the experimental observation by considering the chemical interactions alone as fluid flow decays much faster than chemical fields in a Hele-Shaw cell.

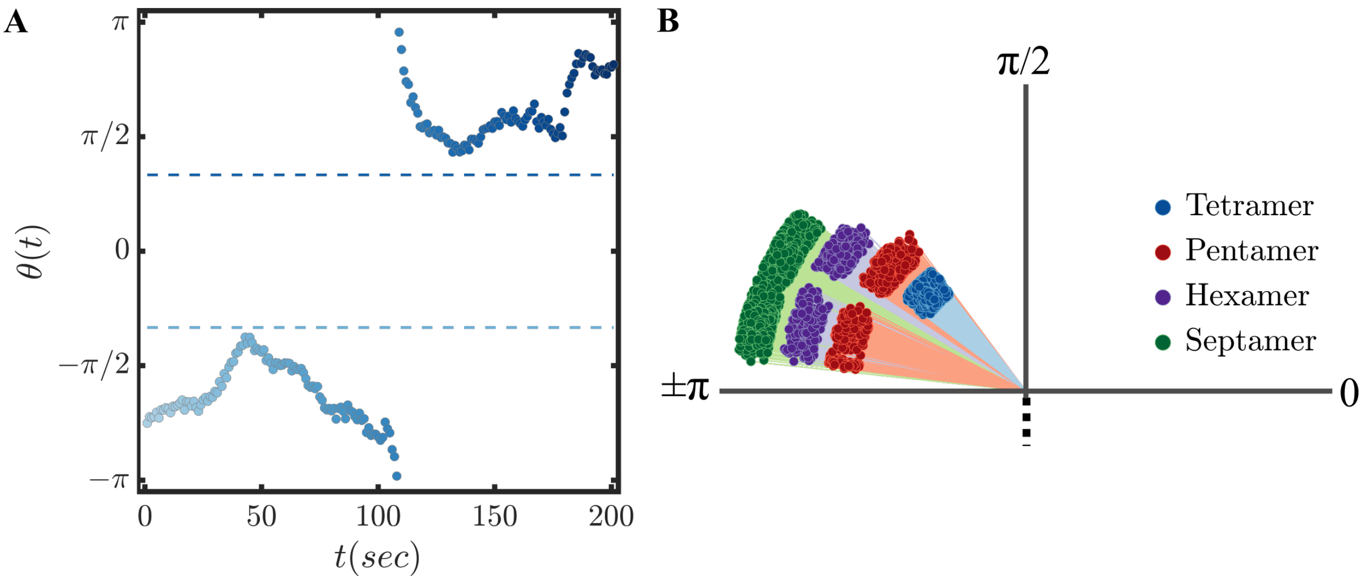

Figure S5. **(A)** Bond Angle of the Nonamer as a function of time; **(B)** Bond angle measured for different active polymer chain lengths ($N = 4$ to $N = 7$)

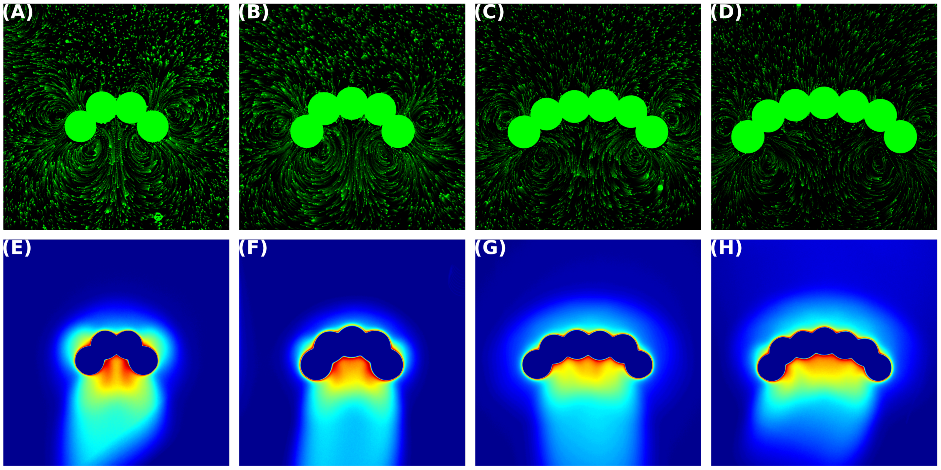

Figure S6. Shows hydrodynamic flow fields and chemical fields measured using a fluorescence microscopy. **(A)** to **(D)** (*left to right, top panel*) are the experimentally measured flow fields of different length active chains (N = 4,5,6,7). Similarly, **(E)** to **(H)** (*left to right, bottom panel*) are the experimentally measured chemical fields of different chain lengths

B. Simulation details

Our simulations are performed using a forward Euler method. In total, we have 10 free parameters, which are the time step, dt, radius of colloid, b, the height of the Hele-Shaw cell H, self-propulsion speed per particle v_s, diffusion constant of filled micelles D_c, translational and rotational susceptibilities, $\tilde{\chi}_t$ and $\tilde{\chi}_r$, with $\tilde{\chi}_i = \frac{\chi_i \lambda_e}{\pi H}$ with λ_e the emission rate of micelles; and the inter-monomer spring constant k_{sp}.. Our choice of parameters are summarized below.

d	dt	b (μm)	H (μm)	v_s ($\mu m/s$)	D_c ($\mu m^2/s$)	$\tilde{\chi}_t$ ($\mu m^3/s^2$)	$\tilde{\chi}_r$ ($\mu m^3/s^2$)	k_{sp} ($\mu m/s^2$)
2	0.01	25	$2b$	10.9	2.5×10^3	1.4×10^4	1.74×10^4	175

Table I. We simulate the dynamics of active polymer chains by numerically integrating Eq.(1) of the main text using an explicit Euler method and the parameters listed above.

In our simulation, we have ignored Brownian motion of the particles, as they are sub-dominant to the active self-propulsion, as we now show. The typical active force due to the self-propulsion of the particle: is $\sim 6\pi\eta b v_s \sim 3.5 \times 10^{-12}$N, where we have used $\eta = 10^{-3} Pa.s.$ On the other hand, the typical Brownian force is of the order $\sim k_B T/b \sim 10^{-16}$ N. We also note that the rotational diffusion can be ignored as can be seen from the mean-squared displacement, obtained from the experimental data, which does not go to diffusive regime even after 10^2s.

Parameters, such as, b, v_s, H are same as in the experiment. $\tilde{\chi}_t$ and $\tilde{\chi}_r$ are chosen to match time-scales in the experimental dynamics of Fig. 2, while k_{sp} is kept large enough so as to mimic the freely joined nature of the polymer using biotin-streptavidin chemistry during the numerical simulation. We note that we have chosen a value of D_c in the simulations, which is larger than those typically measured in experiments on active droplets. This higher value is required to reproduce experimental phenomenology since our minimal model assumes the droplets as point particles, whilst we also ignore the advection of the chemical field due to fluid flow. All the simulations are performed in an unbounded two-dimensional space using custom Python code built on top of a core written in Cython.

C. Chemical field and bond angles

Here, we display further snapshots from simulations of our model for selected values of N. This is shown in Fig.S7 **A**. The scalar chemical field c from our model is displayed on the top most panel. In Fig.S7 **B** we plot the dynamics of β_{in} and β_{out}, which is defined in the inset (color coded). Note that for the $N = 8$ case, there are 6 angles within the chain, and by symmetry only 3 distinct angles.

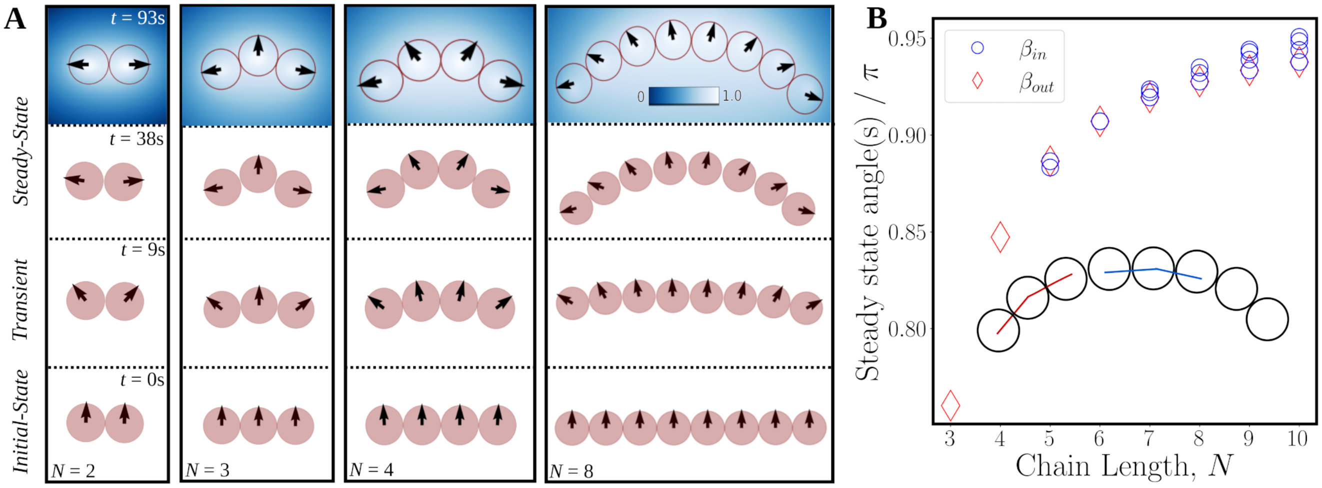

Figure S7. **A** shows snapshots for (*left to right*) $N = 2$, $N = 3$, $N = 4$, and $N = 8$ respectively. Simulation times are shown in the inset. The scalar field c is shown in the topmost row, with color bar in inset. We note that the final steady-state, a stable C-shaped chain, is independent of initial conditions. See video SV17. Panel **B** shows the distribution of unique bond angles along the chain, labelled here as β. The inset shows the respective definitions for β_{in} (blue) and β_{out} (red) respectively.

D. Curvature computation

As a measure of the positional rigidity of the chain, we presented curvature plots in the main text (Figures 2 and 3). To compute the curvature of the chains, we use the Monge representation of the curvature [4], given by

$$\kappa = \nabla \cdot \left[\frac{\nabla h}{\sqrt{1 + |\nabla h|^2}} \right] \tag{S3}$$

where h is the height function of the chain. In our case, this is simply reduced to $\partial_{r_\perp} \left[\frac{\partial_{r_\perp} r_\parallel}{\sqrt{1+|\partial_{r_\perp} r_{||}|^2}} \right]$, where $r_\parallel$ and $r_\perp$ are the coordinates parallel and perpendicular to the direction of propulsion of the chain. An example of the time evolution of κ is shown in Fig S8 below, showing the growth in stiffness of the chain as the rigid "C" shape is attained.

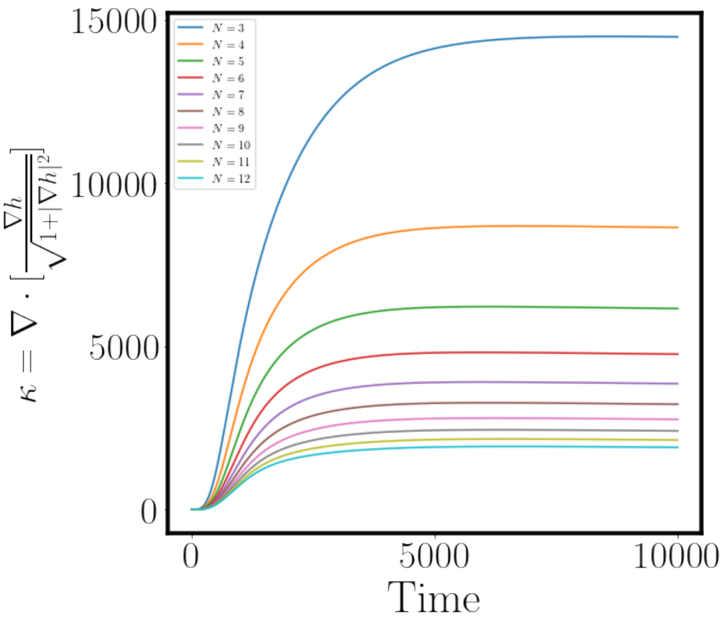

Figure S8. Time evolution of curvature κ of the chain for different values of N starting from a straight configuration as shown in Fig.S7 **A**. A fixed value of κ is obtained for each N in the steady-state as the chain propels in a stable C-shape. See movie SV5.

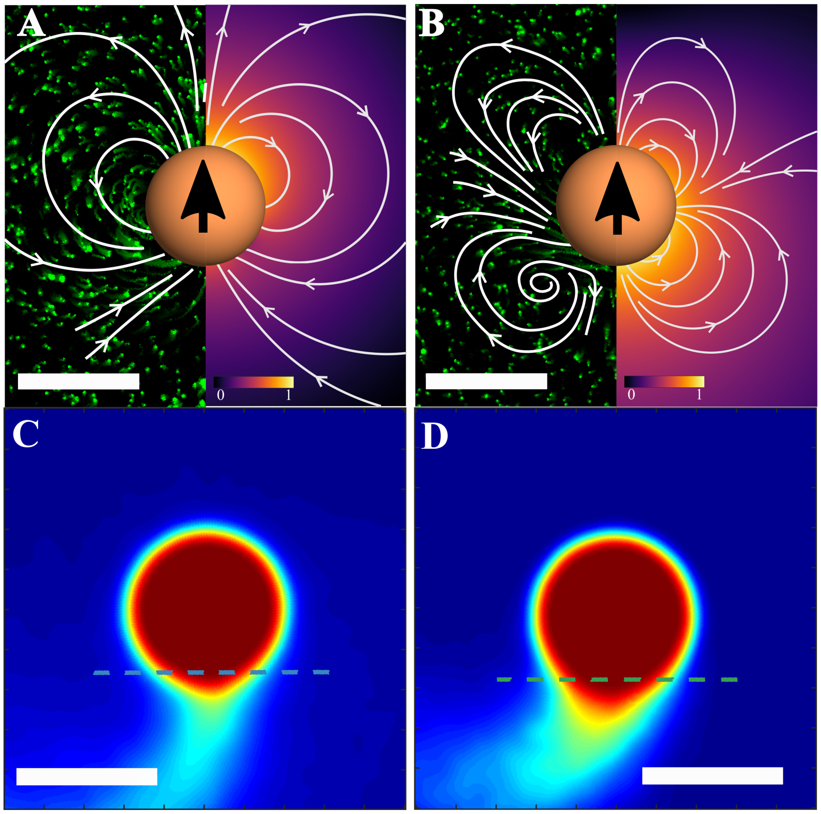

Figure S9. Steady state flow fields and chemical fields of monomer. (**A**) and (**B**) are the “pusher" type of squirmer [5] at $\phi = 0$ and $\phi = 0.2$. (**C**) and (**D**) are the corresponding chemical fields of the monomer, the chemical field intensities are measured along the line shown in the images.

IV. SUPPLEMENTARY VIDEOS

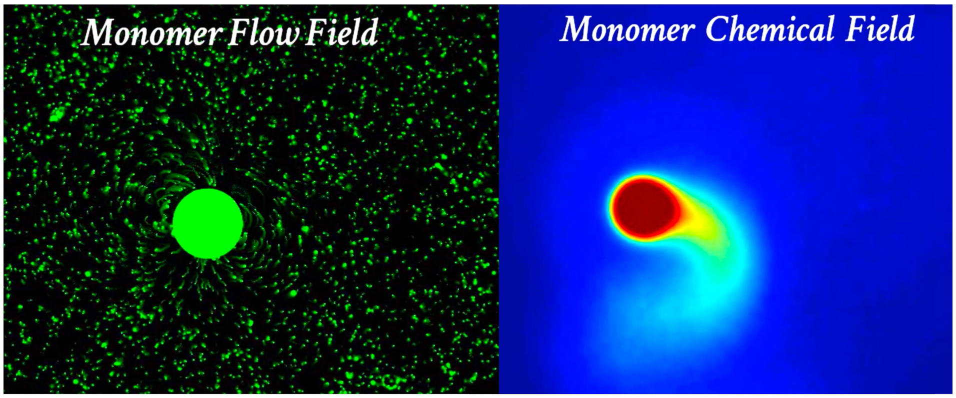

Figure S10. **SV1**: Hydrodynamic field and chemical field of a monomer in a quasi two-dimensional confinement of height, h $\sim 50\ \mu m$.

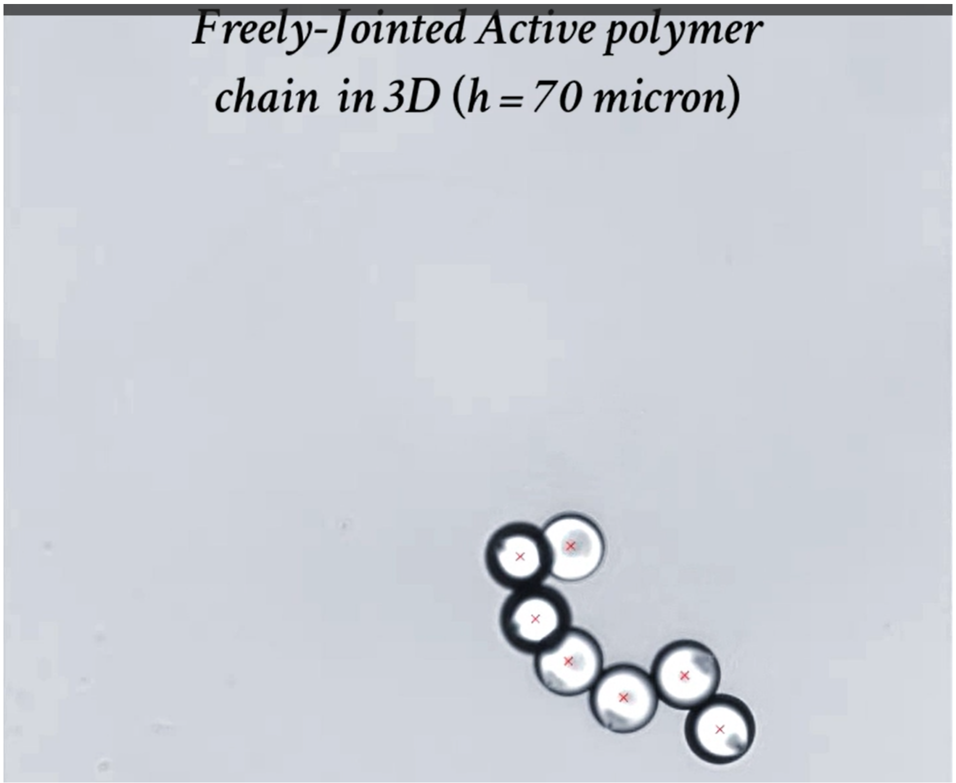

Figure S11. **SV2**: Freely-jointed nature of active polymer chain ($N = 7$) in a three-dimensional confinement (weak confinement).

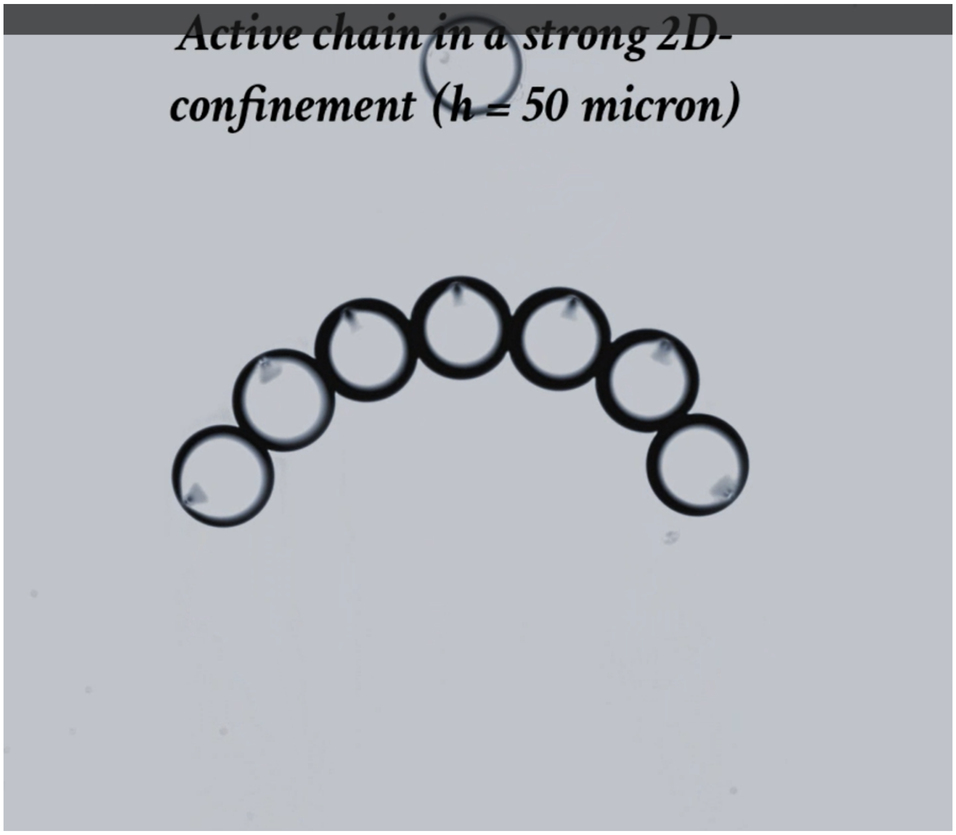

Figure S12. **SV3**: Rigid and stereotypic nature of active polymer chain (N = 7) in a strong 2D confinement.

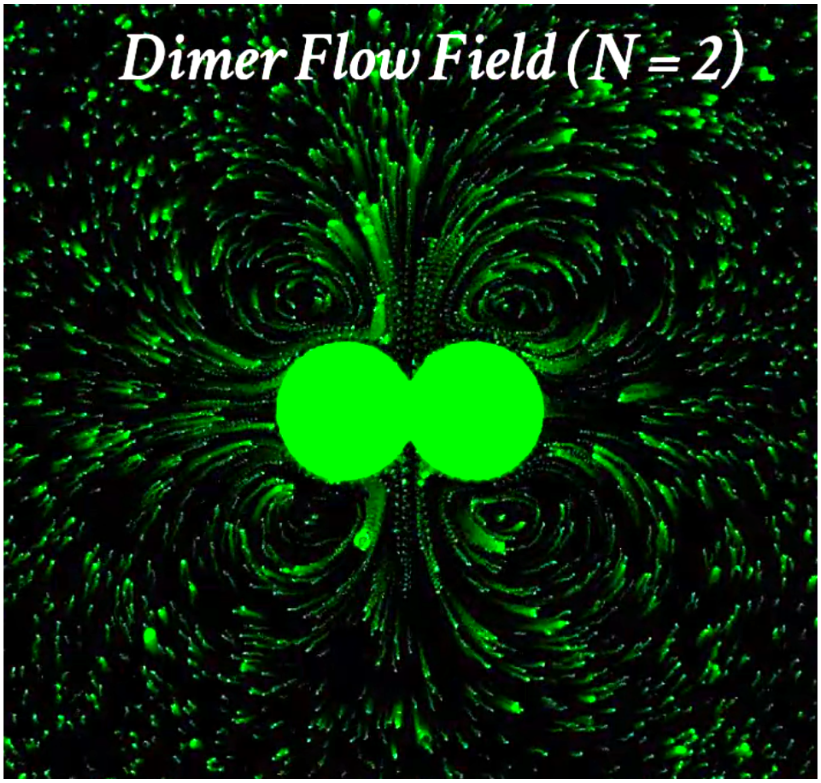

Figure S13. **SV4**: Hydrodynamic field of a dimer (N = 2)

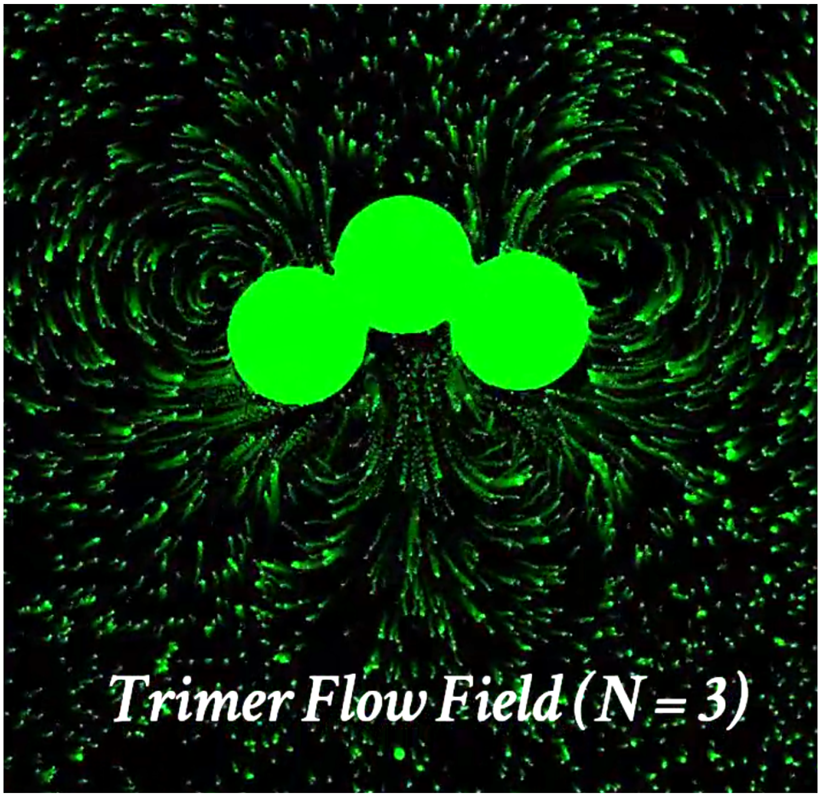

Figure S14. **SV5**: Hydrodynamic field of a 3-mer ($N = 3$)

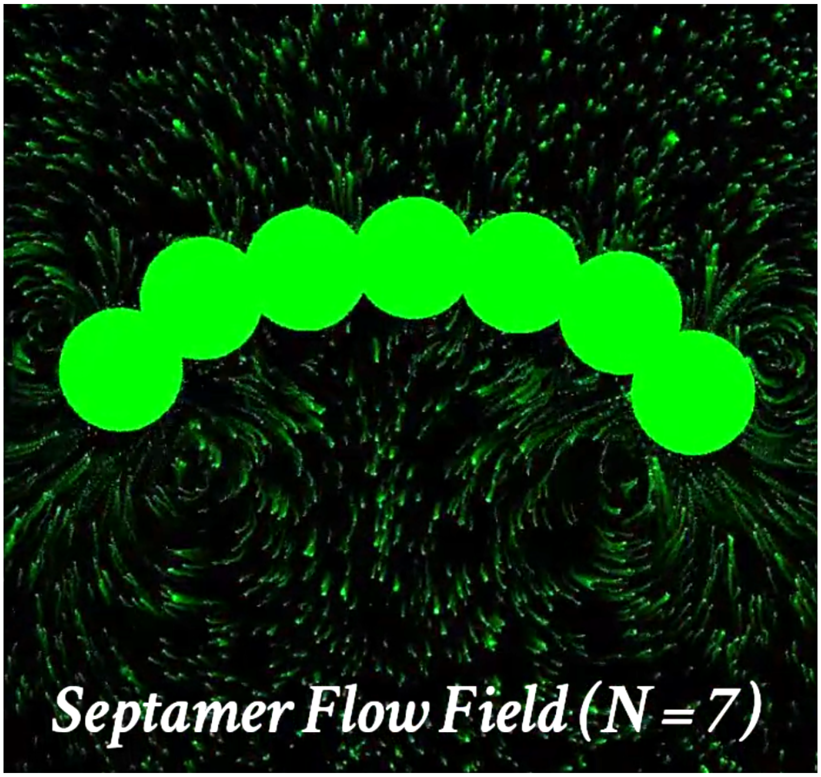

Figure S15. **SV6**:Hydrodynamic field of a 7-mer ($N = 7$)

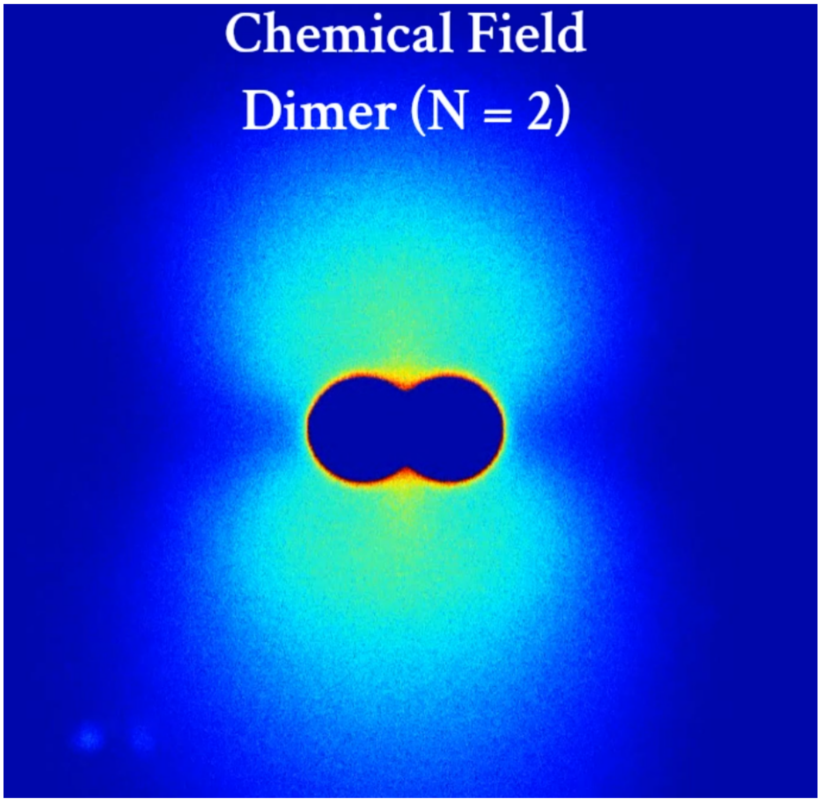

Figure S16. **SV7**: Chemical field of a dimer ($N = 2$)

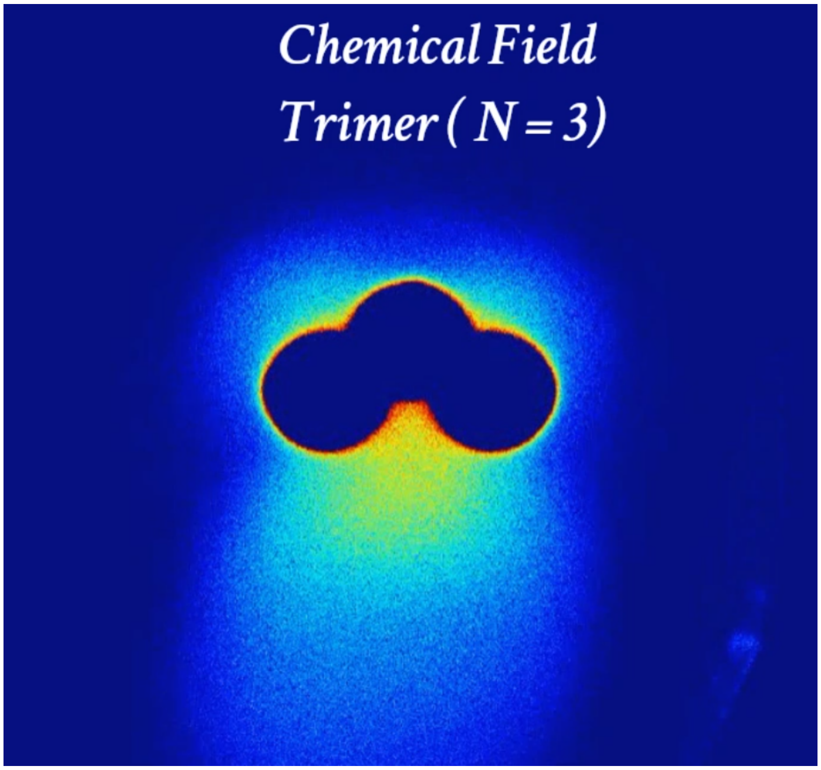

Figure S17. **SV8**: Chemical field of a 3-mer ($N = 3$)

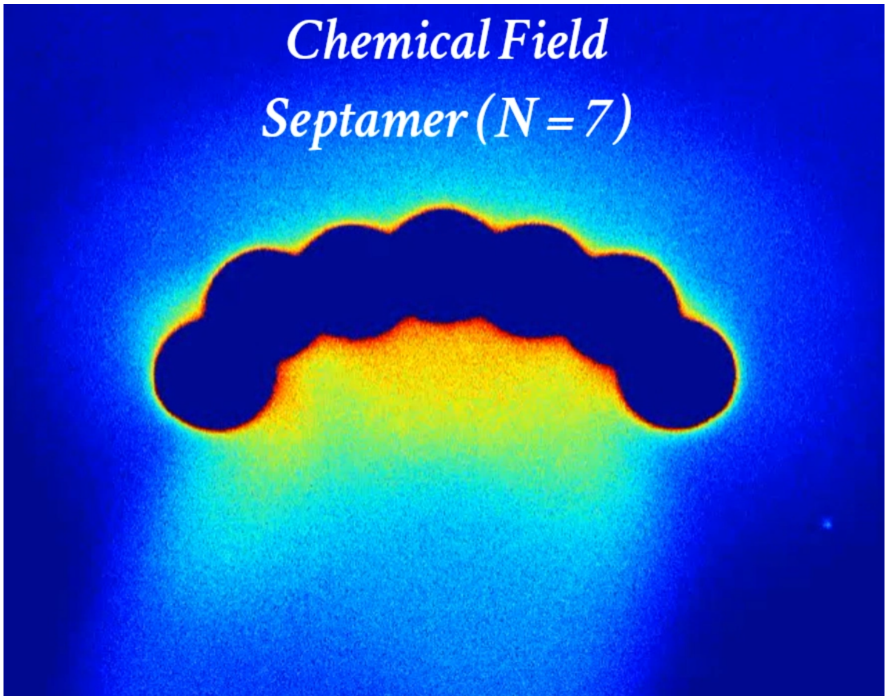

Figure S18. **SV9**: Chemical field of a 7-mer ($N = 7$)

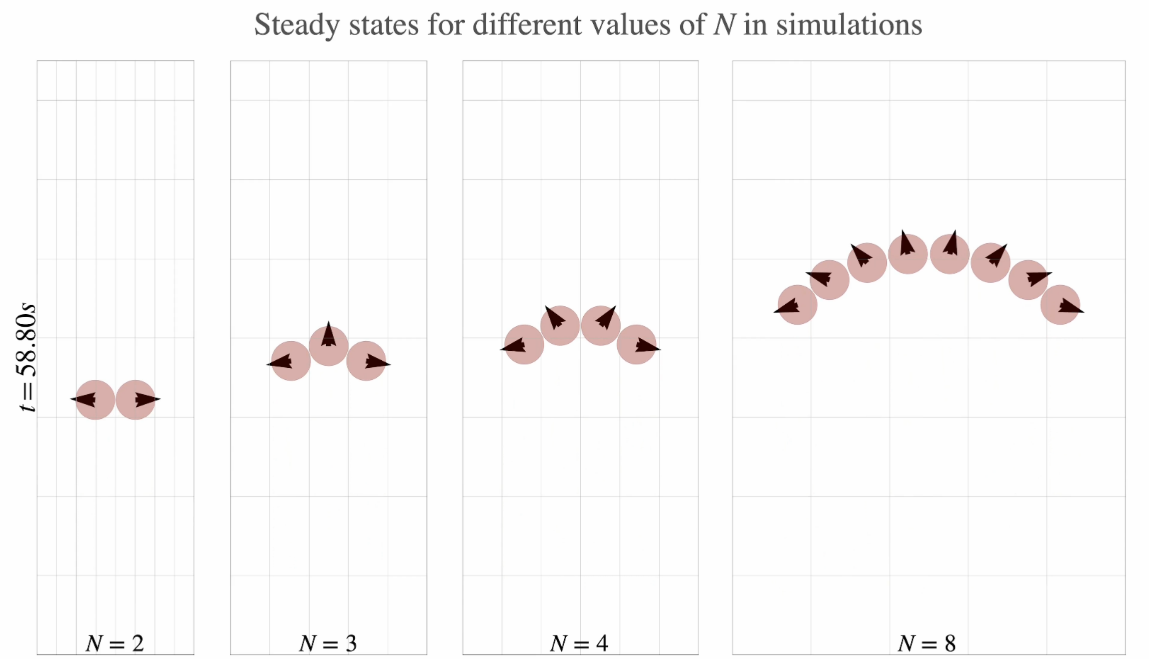

Figure S19. **SV10**: Self-propulsion dynamics of active polymer chains using simulations of our minimal model ($N = 2$, $N = 3$, $N = 4$, and $N = 8$)

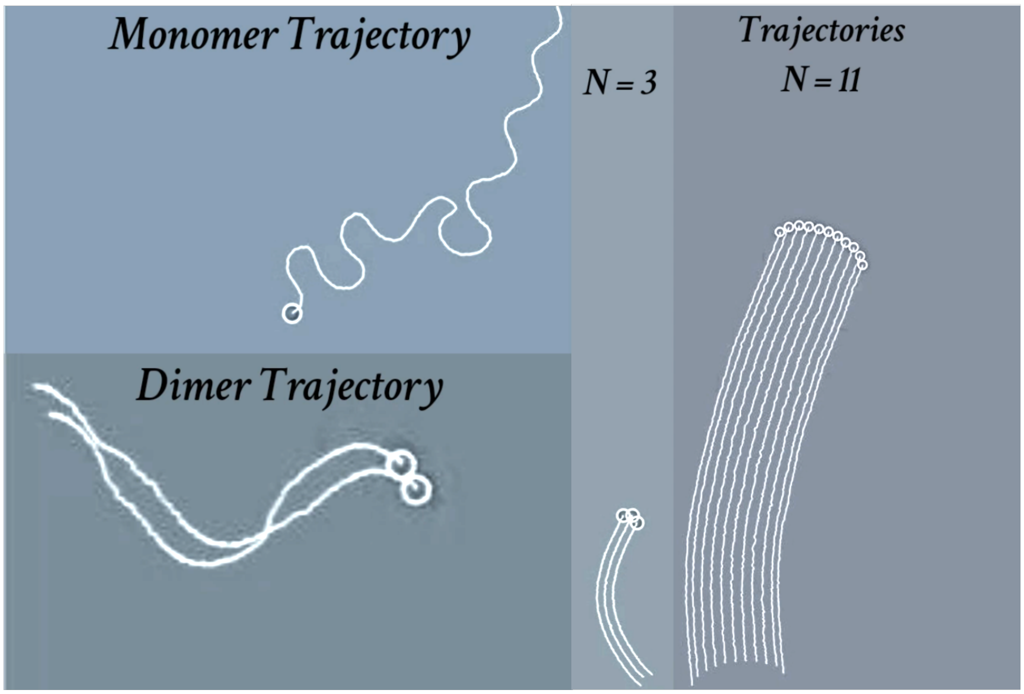

Figure S20. **SV11**: Trajectories of self-propelling active monomer (N = *1*) and other active assemblies (N = *2, 3 and 11*)

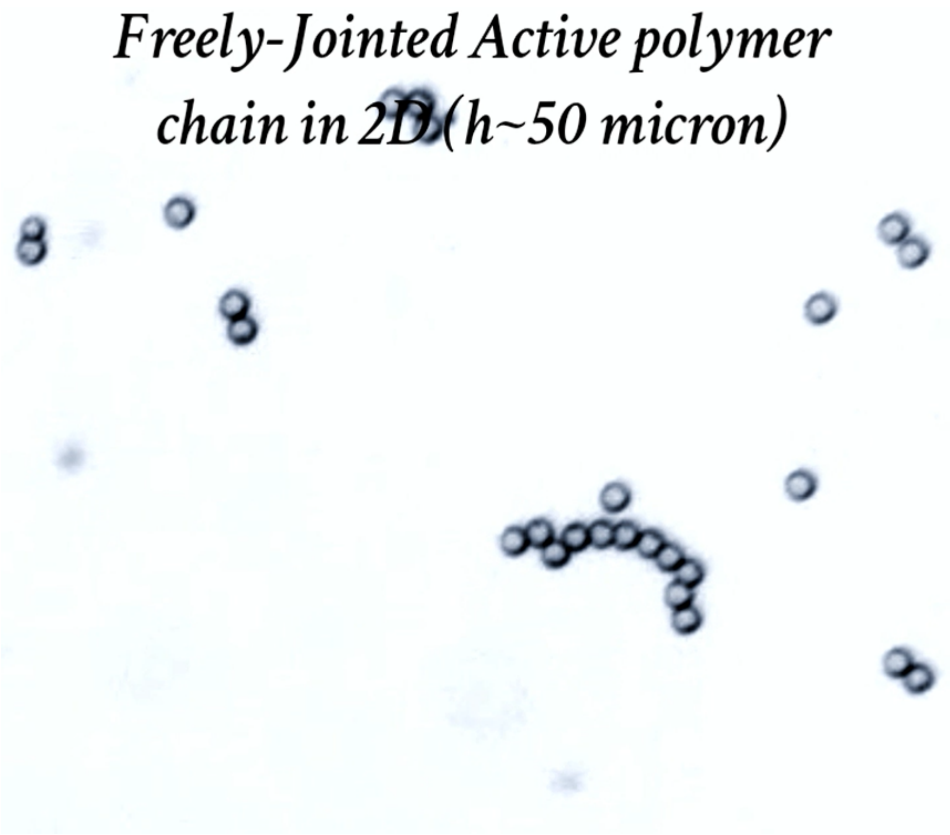

Figure S21. **SV12**: Freely-jointed active polymer chain (N = *11*) becomes rigid in strong 2D confinements and adopts stable C- shape configuration, but destabilised by the collision with the assemblies and monomers present in the surrounding medium. Chain exhibits flexibility and metastable configurations but tends to stabilise the stable C configuration in a quasi two-dimensional confinement.

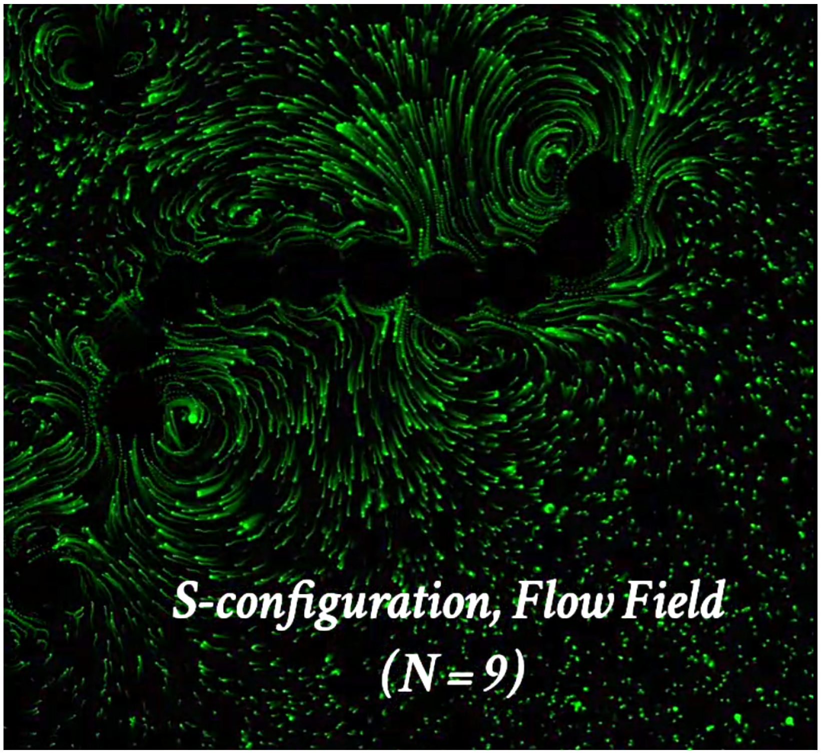

Figure S22. **SV13**: Hydrodynamic field of active polymer chain in a metastable S-configuration ($N = 9$)

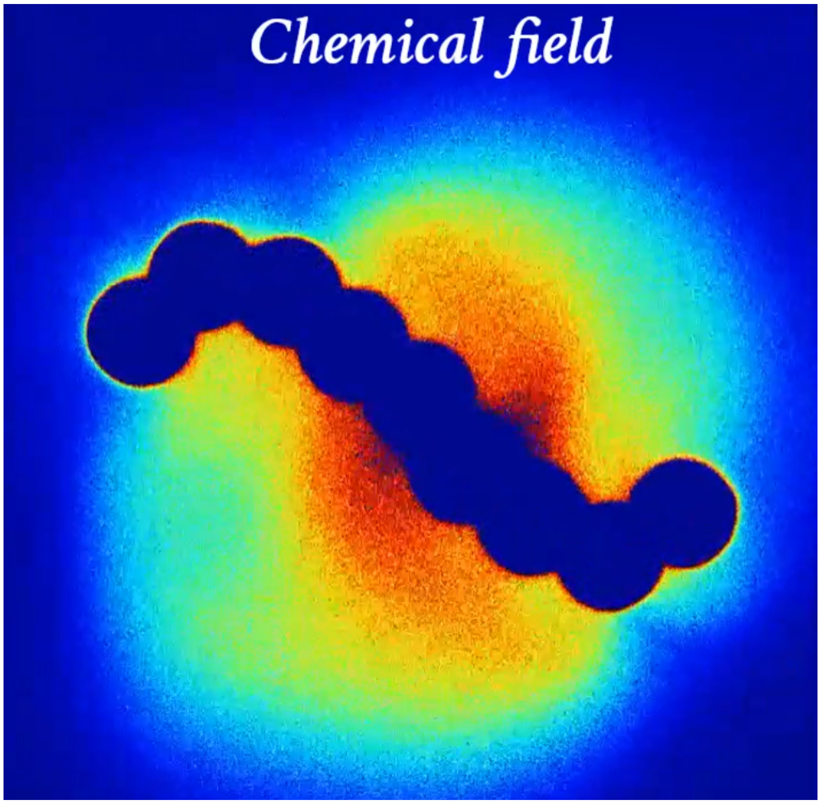

Figure S23. **SV14**: Chemical field of active polymer chain in a metastable S-configuration ($N = 9$)

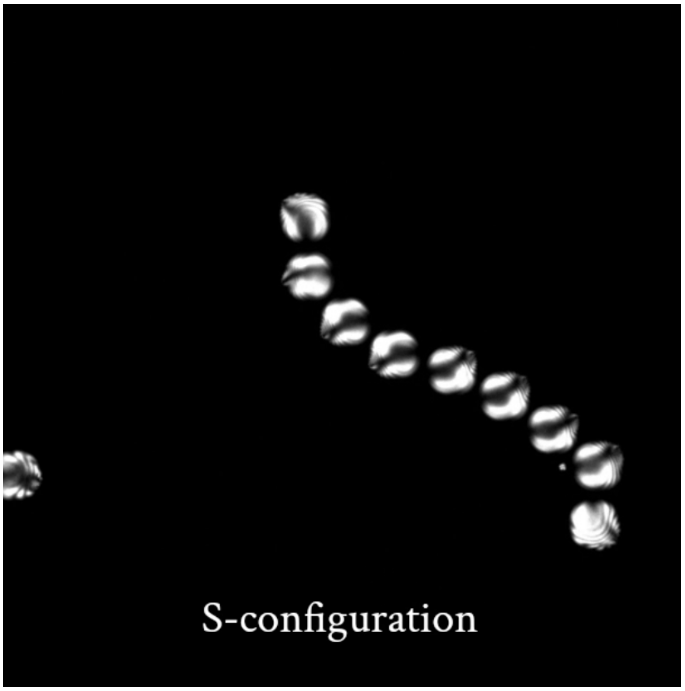

Figure S24. **SV15**: Transition of active chain ($N = 9$) from unstable S-configuration to a stable C-configuration shown in experiments

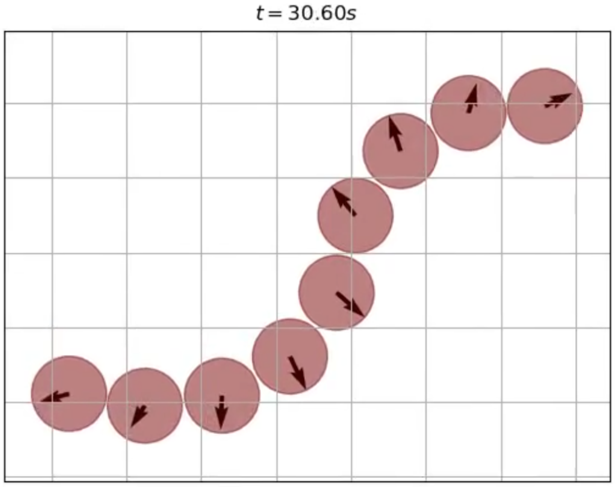

Figure S25. **SV16**: Transition of active chain ($N = 9$) from unstable S-configuration to a stable C-configuration shown in simulation.

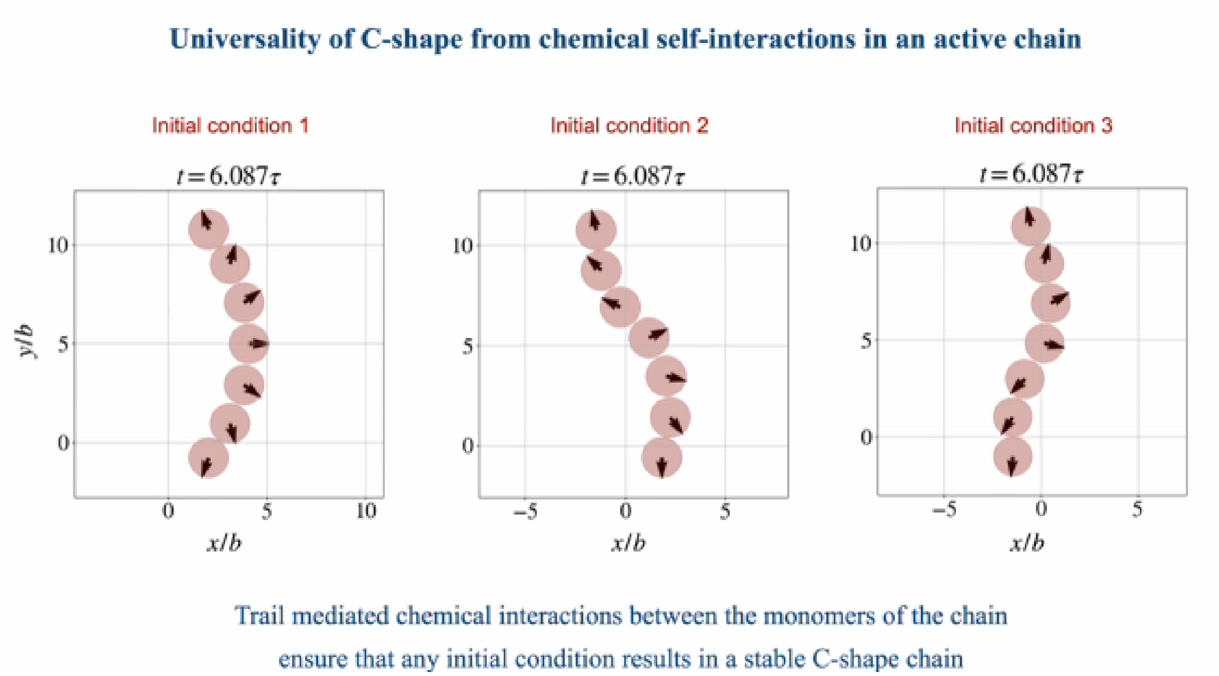

Figure S26. **SV17**: Simulations results show that the emergent steady-state is independent of the initial conditions for a chemically interacting active chain $(N = 7)$.

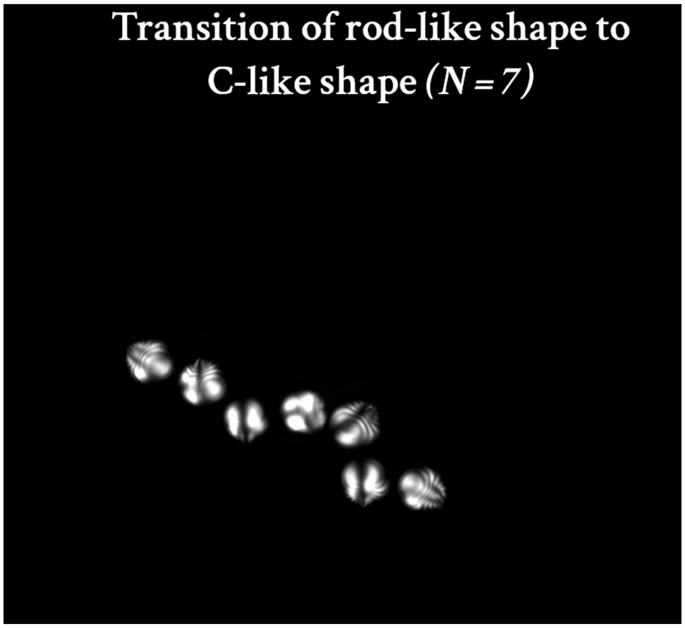

Figure S27. **SV18**: Experimental results show the transition of initial random rod-like shape to a C-like steady state $(N = 7)$.

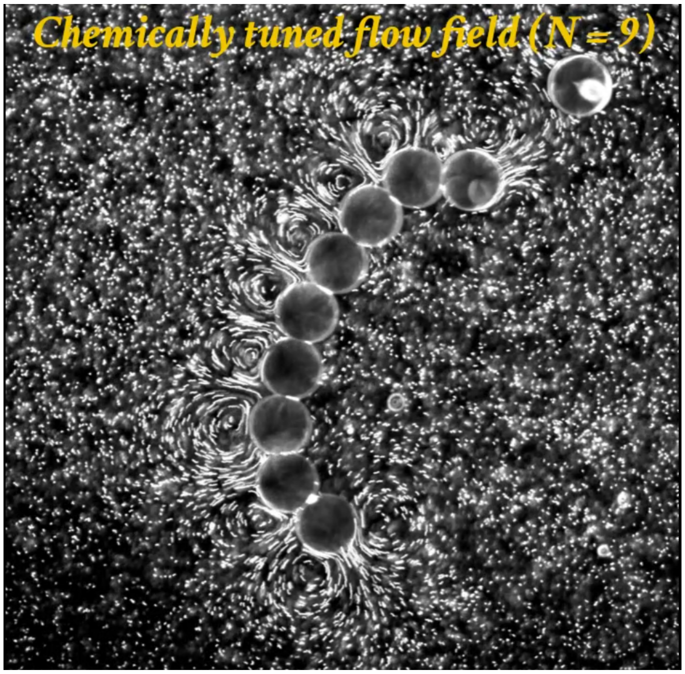

Figure S28. **SV19**: Oscillations in the propulsion dynamics of a trimer ($N = 3$) in a chemically tuned environment with $\Phi = 0.7$ oil-filled micellar solution in a strong 2D confinement. The auto-chemorepulsive interactions of trimer with self-generated chemical field generating oscillations are captured under fluorescence microscopy.

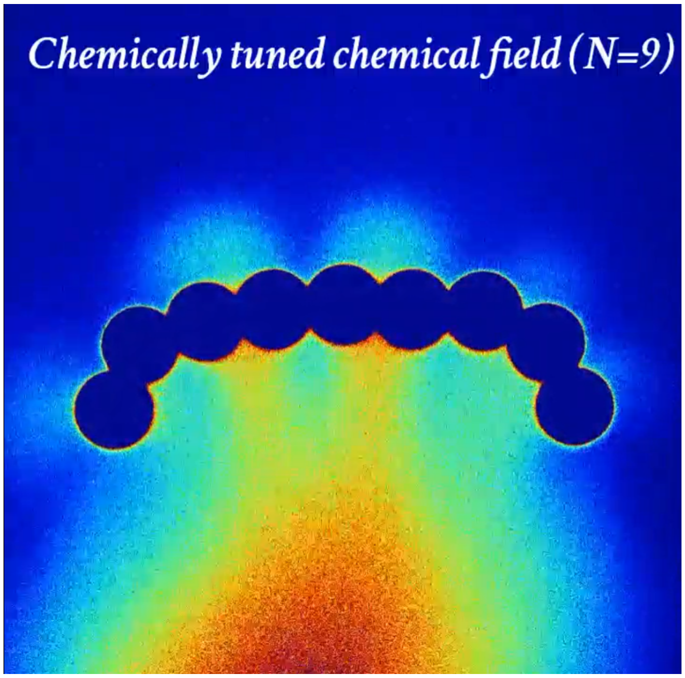

Figure S29. **SV20**: Flow fields ($N = 9$) in a chemically tuned environment with $\Phi = 0.7$ oil-filled micellar solution in a strong 2D confinement.

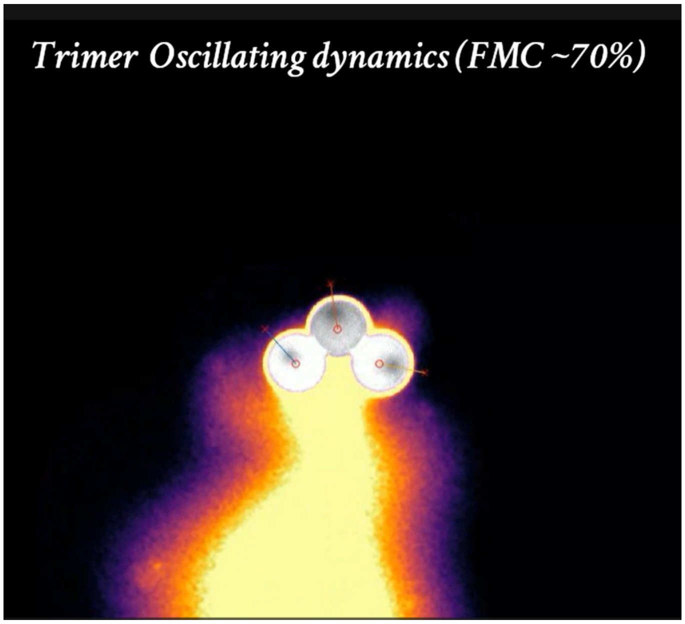

Figure S30. **SV21**: Chemical field ($N = 9$) in a chemically tuned environment with $\Phi = 0.7$ oil-filled micellar solution in a strong 2D confinement.

[1] M. K. Kim, Jiyeon and S.-Y. Par, ACS Appl. Mater. Interfaces **5**, 13131 (2023).
[2] W. Thielicke and R. Sonntag, J. Open Res. Software **9**, (2021).
[3] V. N. P. Gilpin, William and M. Prakash, J. Exp. Biol. **220** , 3411 (2021).
[4] W. Helfrich, Zeitschrift fÃŒr Naturforschung c **28** , 693 (1973).
[5] R. E. Goldstein, Annu. Rev. Fluid Mech. **47** , 343 (2015).
