# Supplementary material for: Emergent dynamics due to chemo-hydrodynamic self-interactions in active polymers
Source: Nat Commun. 2024 Jun 8;15:4903. doi: 10.1038/s41467-024-49155-7 (PMC11162426; doi:10.1038/s41467-024-49155-7)
Supplement: Supplementary file 3 — Description of Additional Supplementary Files [file 41467_2024_49155_MOESM3_ESM.pdf]

# Description of Supplementary Videos

**File Name:** Supplementary Video 1 (SV1)
**Description:** Hydrodynamic field and chemical field of a monomer in a quasi-two-dimensional confinement of height, h ~ 50 μm.

**File Name:** Supplementary Video 2 (SV2)
**Description:** Freely-jointed nature of active polymer chain (N = 7) in a three-dimensional confinement (weak confinement).

**File Name:** Supplementary Video 3 (SV3)
**Description:** Rigid and stereotypic nature of active polymer chain (N = 7) in a strong 2D confinement.

**File Name:** Supplementary Video 4 (SV4)
**Description:** Hydrodynamic field of a dimer (N = 2).

**File Name:** Supplementary Video 5 (SV5)
**Description:** Hydrodynamic field of a 3-mer (N = 3).

**File Name:** Supplementary Video 6 (SV6)
**Description:** Hydrodynamic field of a 7-mer (N = 7).

**File Name:** Supplementary Video 7 (SV7)
**Description:** Chemical field of a dimer (N = 2).

**File Name:** Supplementary Video 8 (SV8)
**Description:** Chemical field of a 3-mer (N = 3).

**File Name:** Supplementary Video 9 (SV9)
**Description:** Chemical field of a 7-mer (N = 7).

**File Name:** Supplementary Video 10 (SV10)
**Description:** Self-propulsion dynamics of active polymer chains using simulations of our minimal model (N = 2, N = 3, N = 4, and N = 8).

**File Name:** Supplementary Video 11 (SV11)
**Description:** Trajectories of self-propelling active monomer (N = 1) and other active assemblies (N = 2, 3 and 11).

**File Name:** Supplementary Video 12 (SV12)
**Description:** Freely-jointed active polymer chain (N = 11) becomes rigid in strong 2D confinements and adopts stable C- shape configuration, but destabilised by the collision with the assemblies and monomers present in the surrounding medium. Chain exhibits flexibility and metastable configurations but tends to stabilise the stable C configuration in a quasi two-dimensional confinement.

**File Name:** Supplementary Video 13 (SV13)
**Description:** Hydrodynamic field of active polymer chain in a metastable S-configuration (N = 9).

**File Name:** Supplementary Video 14 (SV14)
**Description:** Chemical field of active polymer chain in a metastable S-configuration (N = 9).

**File Name:** Supplementary Video 15 (SV15)
**Description:** Transition of active chain (N = 9) from unstable S-configuration to a stable C-configuration shown in experiments.

**File Name:** Supplementary Video 16 (SV16)
**Description:** Transition of active chain (N = 9) from unstable S-configuration to a stable C-configuration shown in simulation.

**File Name:** Supplementary Video 17 (SV17)
**Description:** Simulations results show that the emergent steady-state is independent of the initial conditions for a chemically interacting active chain (N = 7).

**File Name:** Supplementary Video 18 (SV18)
**Description:** Experimental results show the transition of initial random rod-like shape to a C-like steady state (N = 7).

**File Name:** Supplementary Video 19 (SV19)
**Description:** Oscillations in the propulsion dynamics of a trimer (N = 3) in a chemically tuned environment with $\Phi = 0.7$ oil-filled micellar solution in a strong 2D confinement. The auto-chemorepulsive interactions of trimer with self-generated chemical field generating oscillations are captured under fluorescence microscopy.

**File Name:** Supplementary Video 20 (SV20)
**Description:** Flow fields (N = 9) in a chemically tuned environment with $\Phi = 0.7$ oil-filled micellar solution in a strong 2D confinement.

**File Name:** Supplementary Video 21 (SV21)
**Description:** Chemical field (N = 9) in a chemically tuned environment with $\Phi = 0.7$ oil-filled micellar solution in a strong 2D confinement.
